# Bridging the activation gap: a physical activity intensity scale informed by elite athletes yet inclusive for musculoskeletal pain patients

**DOI:** 10.3389/fspor.2025.1641818

**Published:** 2025-11-27

**Authors:** Matthew Fernandez, Stephen Seiler

**Affiliations:** 1 School of Health, Medical and Applied Sciences, CQUniversity, Brisbane, QLD, Australia; 2 Department of Sport Science and Physical Education, University of Agder, Kristiansand, Norway

**Keywords:** physical activity, exercise, musculoskeletal, pain, intensity zones

## Introduction

Physical activity (PA) is a cornerstone of public health, with the World Health Organization's (WHO) PA/sedentary behavior (SB) guidelines recommending at least 150 min per week of moderate or 75 min per week of vigorous activity per week, plus resistance training (1). Despite ongoing promotion of PA/SB guidelines, there has been a global increase in population estimates of insufficient PA levels from 2000 to 2022, rising from 23% to 31% (2).

Musculoskeletal disorders like osteoarthritis, low-back pain and neck pain are a major public health concern (3). While PA is generally safe, people with musculoskeletal pain may perceive it as threatening, leading to avoidance and inactivity (4). Pain no longer reflects tissue damage but a learned, sensitized neural pattern amplifying the ongoing pain experience (5). Prolonged inactivity also worsens health. For instance, chronic inflammation and weight gain—chiefly in osteoarthritis—raises cardiovascular risk, creating a tough cycle for those in pain.

In the 1990's, PA/SB guidelines shifted from vigorous exercise for fitness targeting moderate-intensity activities of daily living (ADLs) to stay healthy (6). Recently, new 24 h movement guidelines emphasize total PA across the day, factoring in sedentary time and sleep (7)., and along with wearable technology, has attempted to further promote and leverage the benefits of light as well as intense, but short PAs (8). In this piece, we propose combining two intensity strategies to support inclusive PA promotion—supporting healthcare professionals in promoting movement for those with musculoskeletal disorders and/or chronic pain.

**Figure 1:**
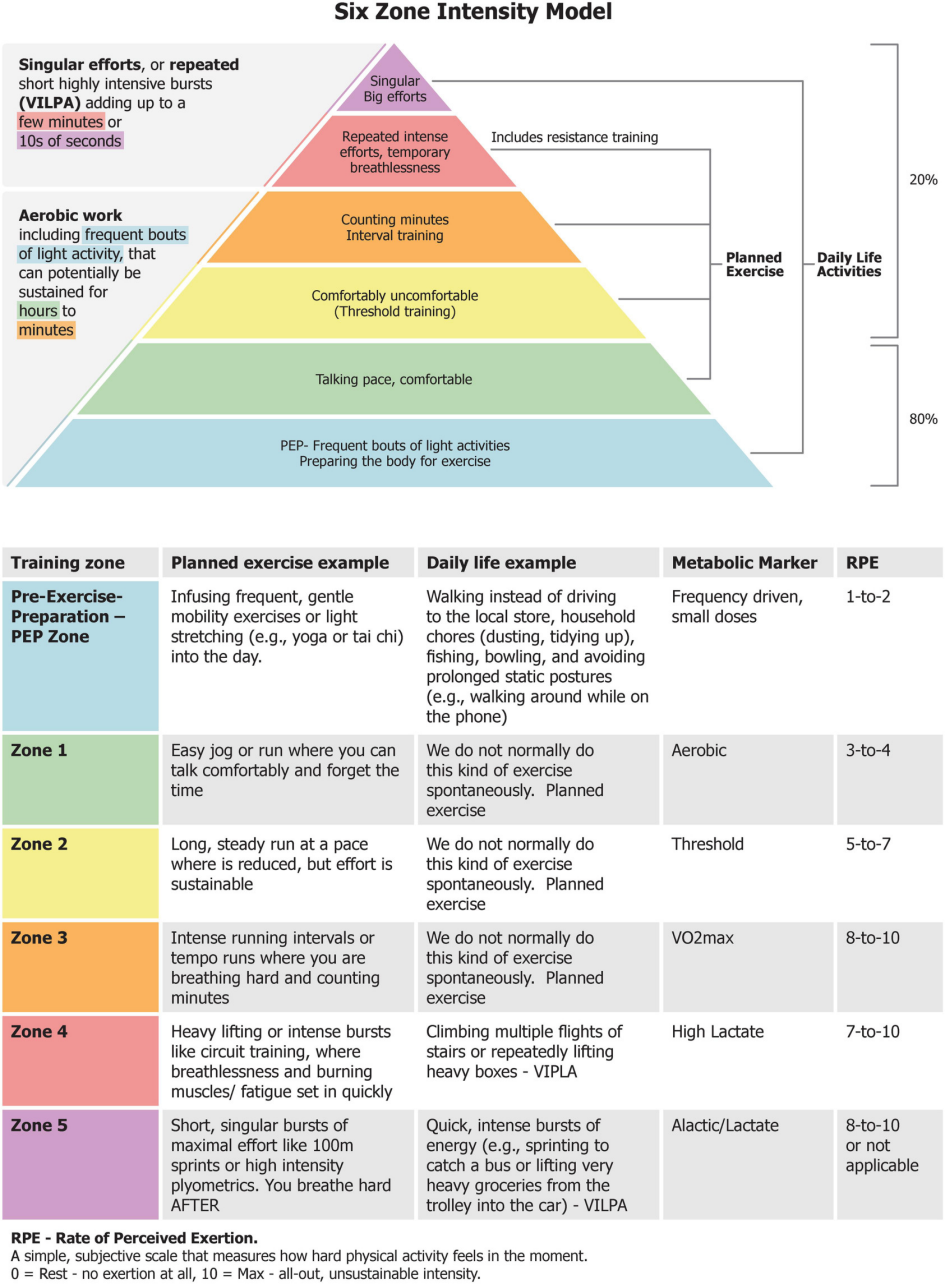
Illustrates the six training zones that guide the prescription and monitoring of physical activity for individuals with musculoskeletal disorders, while also serving a broader purpose: enabling people without prior training - or those returning from injury - to safely and confidently adopt a structured exercise routine that supports everyday physical demands without incident or limitation.

Drawing from two decades of research in well trained to elite athletes, we aim to categorize PA intensity within the framework of the PA/SB recommendations. We propose a non-structured dominance of low or light-intensity PA that resonates for not only sedentary, often “PA fearful” individuals with musculoskeletal disorders, but also for athletes recovering from injury, and others with chronic conditions. We propose an “Activation” or pre-exercise-practice (PEP) zone to complement the traditional three physiologically demarcated “aerobic” exercise intensity zones—zone 1-low aerobic, zone 2-moderate/threshold and zone 3-high intensity/VO2max (9)**.** Then two additional zones we add on top of these includes zone 4—“typical” strength training and other brief (repeated) short bursts of vigorous activity leaving one breathless and zone 5—“maximal efforts” like carrying a heavy box up several flights of stairs. Essentially, we are proposing a “universal intensity scale” that combines both very light and very heavy ADLs combined with established intensity zones used during typical “planned exercise/training” (10). Importantly, these training zones (Figure 1) are generalizations; they regulate exercise stress using robust and accessible physiological markers like breathing frequency, heart rate and perceived exertion (11). These intensity zones are based on the experiences of coaches and athletes who train and perform daily in the endurance arena. Due to individual variability in perceived effort, physiological responses (e.g., Rate of Perceived Exertion—RPE) and fatigue, universal standards for defining training intensity zones remain limited. Our proposed approach utilizes these generalizations to incorporate PA for people with musculoskeletal disorders.

## The “80/20” ratio training intensity—80%

The proposed “80/20” ratio in endurance training refers to the consistent finding across endurance sports that most of these kilometers, miles or minutes, are performed at a comfortable pace. Elite endurance athletes competing over distances from 4 min to >4 h, regularly complete at least 80% of their training days at a low relative intensity and high volume, within a training rhythm that is interspersed with (and allows recovery from) higher intensity training. This higher intensity comprising 20% (12) is important but more is not better. It's a proven basic approach for endurance athletes and we propose extending it to a wider population, especially for those who are injured, insufficiently active, or sedentary. Hence, 80% of PA effort will be foundational work, initially emphasizing frequency that is comprised of consistent, accumulated light endurance-based PA, then gradually increasing session duration, building confidence and capacity for occasional high-intensity aerobic days. This progression not only improves durability through low force aerobic activity—stimulating respiratory and cardiovascular systems, but strengthens and remodels soft tissue like tendons, joint, bones and ligaments through gradual neuromuscular adaptation, without excessive mechanical stress or induced fatigue (13). For athletes, these may serve as “easy” sessions or a “base” training session early in the season or “recovery” between intense sessions. Recent studies using wearables suggest that light PA is beneficial for health (14), with light activity needing 3–4x more time than moderate-to-vigorous PA to reduce disease risk (1). Prioritizing frequency, duration, and total volume, with careful integration of highly intense workouts is a well-established formula that has emerged from studies of elite endurance athletes, but “scales down” to active people of all ages and fitness levels (15).

## The “80/20” ratio training intensity—20%

Vigorous PA interval training is typically comprised of repeated “bouts” of high-intensity efforts lasting ∼30 s to several minutes, like sprinting, and alternated with of lower-intensity recovery (16). There does not seem to be any, one, “best” interval training prescription. High intensity interval training or HIIT (zone 3, Figure 1) is effective, but shows fast plateau effects (17). Further, training in the “threshold zone” (Zone 2, Figure 1) is also very demanding, but the intensity is slightly lower, and the accumulated work duration is markedly longer. Combined, training in Zone 2 and 3 make up the remaining 20% of the ∼80/20 intensity distribution seen in successful endurance athletes and are a staple in athletes' training programs (16). A handful of carefully selected at or near maximal effort sprints of just a few seconds, or longer interval training efforts lasting a few minutes (performed above moderate intensity PA) can both enhance cardiorespiratory fitness when the baseline is low (18). The substantial rates of force production and peak forces activates anaerobic energy pathways and intense neuromuscular activation (13). Further, the potent neuromuscular stimuli are similar in nature (though not in structure) to elements of strength or power training, and may counteract the progressive decline of muscle mass, voluntary muscle activation and eventual atrophy (19).

## Discussion: a new zone for musculoskeletal disorders?

The “80/20” ratio of training intensity guides volume distribution between lighter- and harder-intensities and helps introduce an additional training zone that explicitly targets those with musculoskeletal disorders. Physical inactivity significantly increases the risk of morbidity and mortality (1). Moreover, sedentary behaviours are linked with musculoskeletal disorders, and the typical “Zone 1” threshold (e.g., a 30 min conversational jog) may still be too intense for people experiencing chronic pain. We therefore propose a “PEP” zone as a first recommendation of light PA intensity level, to facilitate essential adaptations for people with musculoskeletal disorders (Figure 1). With no demands for intensity compliance, targeted goals or pressure, PEP proposes a safe and progressive way to engage in PA via small, frequent and cumulative movement doses without exacerbating symptoms. Examples of light, unstructured ADLs include casual walking, fishing, gardening or playing with children, and household chores like vacuuming or hanging laundry.

For individuals with low PA levels, small steps toward moving more can have lasting health benefits. For the injured, deconditioned or recovering (athletic) population, this PEP zone can be a gateway to support the health of surrounding tissues. Significant health benefits accrue when transitioning from complete inactivity to a small amount of PA (20). The PEP zone acknowledges this critical and often difficult transition from inactivity-to-movement, closely mirroring the recently proposed “Very Low” intensity category found in emerging expert consensus frameworks, which highlight the health benefits of any movement—even in the absence of a clear physiological marker (21). As such, purposefully completing frequent, short bouts of light PA is a simple way to gradually build PA sustainability, with duration and intensity to follow e.g., zone 1—all while building confidence and reducing fear of movement. Arguably, this important strategy helps break the pain-inactivity cycle (22). Often overlooked in PA/SB guidelines, light PA is vital and integrated into our proposed scale via ADLs.

Walking exemplifies PEP and Zone 1. While step goals aren't set in the current PA/SB guidelines, 10,000 steps per day is a popular target (23), whereas ∼7,000 daily steps is linked to strong health benefits (24). Evidence further shows that each 1,000-step increase results in a 15% reduction in all-cause mortality, and a 500-step increase lowers cardiovascular mortality risk by 7% (20). Even 2,500–4,000 steps daily offer measurable benefits (20), reinforcing the WHO's message: “every move counts, but more is better” (1).

Importantly, we acknowledge that the WHO endorse moderate-to-vigorous PA (1). For those with musculoskeletal disorders, reservations can be raised as to whether HIIT-like PA is a realistic and sustainable way to improve health (25). This kind of high intensity work is more palatable in smaller doses achieved in unstructured, practical life settings. Recent evidence shows that vigorous intermittent lifestyle PA (VILPA) lasting up to one or two minutes per day, can significantly lower the risk of chronic disease and premature death (8). Real-world ADLs like quickly climbing stairs two at a time, running to the bus, digging or shoveling in the garden, or carrying heavy groceries to a car parked a fair distance from the store entrance are all examples of VILPA that can provide significant health benefits. If we consider the “80/20” ratio over time, people with musculoskeletal disorders can make real progress by spending sufficient time in the PEP zone through their ADLs with very brief periods spent in Zone 4 and 5, to achieve powerful bursts of PA. The added benefit of clear, recognizable guidelines like the common RPE scales, also help keep those with musculoskeletal disorders within their ideal exertion range (Figure 1).

By using a “PEP and +5 zone model”, we propose a “universal” PA intensity zone continuum to embrace clinical diversity in practice, i.e., real-world applicability and individualization for musculoskeletal disorders. Further, it is applicable to healthy recreational exercisers, competitive age group athletes, and elite performers. We recognize that even elite athletes are susceptible to injury and psychological challenges trying to return to pre-injury fitness (26), hence the continuum includes the PEP zone to facilitate their return to peak performance.

We seek an approach that conforms to epidemiological evidence-based guidelines, while building confidence and reducing injury fears. The universal scale we propose seamlessly integrates “daily life chores and challenges” as health promoting PA (PEP + Zones 4 and 5) alongside “planned” exercise guided by traditional aerobic intensity zones (Zones 1, 2, and 3). In essence, while training zones vary, the body adapts in similar ways, leading to comparable physiological benefits. This reflects the “bow-tie architecture”, where varied zone intensities—including low stress PEP—act as diverse stressor inputs. These inputs converge into a central regulatory hub (the “knot”), which then drives a wide range of beneficial adaptations (27).

The strength of the PEP zone lies in promoting active habits, countering inactivity (e.g., putting on shoes, opening the front door, and taking a walk) and, at least in the beginning, consistency matters more than heart rate zones, lactate thresholds, or fitness gains. The PEP zone also supports well-being, and may help reduce pain-related fear, reshaping avoidance behaviours (28). Notwithstanding, some discomfort may be part of the PEP zone. Fostering trust in the body's recover through gentle, purposeful movements, supports adaptation and may temporarily ease pain (22). As such “hurt doesn't equal harm” is true when building a habit of regular PA through the PEP zone.

Intensity zones are individualized, complementing the population-focused PA/SB guidelines by communicating a common “intensity language”. Researchers, athletes, trainers and coaches have long guided exercise through clear zone instruction (29), a practice now further supported by a standardized intensity framework with aligned effort descriptors aimed at harmonizing terminology across PA, exercise, and sport and human performance disciplines (21). Healthcare professionals can adjust intensity levels for gradual, sustainable progress, boosting adherence and motivation in patients with musculoskeletal disorders. Figure 1 is a quick reference, easy-to-follow educational pyramid that prioritizes small, yet frequent bouts of light intensity PA (PEP zone), followed by longer duration (Zone 1) bouts of PA as a foundation—the 80%—for building tolerance to progressively higher intensities of PA, representing the remaining 20% (Zones 2–5). Healthcare professionals can tailor PA zones to everyone's health, goals, and capacity. For instance, the PEP zone and zone 4 or 5 can typically be met via ADLs with fluctuating intensity. Zones 1–3 typically reflect “planned exercise” at moderate to vigorous-intensity training, aligning with the PA/SB guidelines.

Importantly, our proposed PEP zone—designed to complement traditional exercise intensity zones—remains a theoretical framework. To assess its feasibility and effectiveness, validation through real world implementation in clinical populations is needed, ideally using cohort or pragmatic study designs supported by wearable technology.

## Conclusion

Physical inactivity remains a global health challenge. For people with musculoskeletal disorders, we propose the “80/20” ratio as a clinically and behaviorally useful way to simplify exercise guidance—while aligning with the existing literature. The new PEP zone offers a safe, inclusive and gradual path for people with musculoskeletal disorders to build endurance and strength without fear or strain through practical everyday activity.
